# Trunk's Natural Inclination Influences Stance Limb Kinetics, but Not Body Kinematics, during Gait Initiation in Able Men

**DOI:** 10.1371/journal.pone.0055256

**Published:** 2013-01-30

**Authors:** Sébastien Leteneur, Emilie Simoneau, Christophe Gillet, Yoann Dessery, Franck Barbier

**Affiliations:** 1 Université Lille Nord de France, Lille, France; 2 UVHC, LAMIH, Valenciennes, France; 3 CNRS, UMR CNRS 8201, Valenciennes, France; 4 Centre de Rééducation Fonctionnelle La Rougeville, Saint-Saulve, France; 5 Université Laval, Division de Kinésiologie, Faculté de Médecine, Québec, Canada; University of South Australia, Australia

## Abstract

The imposing mass of the trunk in relation to the whole body has an important impact on human motion. The objective of this study is to determine the influence of trunk's natural inclination - forward (FW) or backward (BW) with respect to the vertical - on body kinematics and stance limb kinetics during gait initiation.

Twenty-five healthy males were divided based on their natural trunk inclination (FW or BW) during gait initiation. Instantaneous speed was calculated at the center of mass at the first heel strike. The antero-posterior impulse was calculated by integrating the antero-posterior ground reaction force in time. Ankle, knee, hip and thoraco-lumbar (L5) moments were calculated using inverse dynamics and only peaks of the joint moments were analyzed. Among all the investigated parameters, only joint moments present significant differences between the two groups. The knee extensor moment is 1.4 times higher (*P*<0.001) for the BW group, before the heel contact. At the hip, although the BW group displays a flexor moment 2.4 times higher (*P*<0.001) before the swing limb's heel-off, the FW group displays an extensor moment 3.1 times higher (*P*<0.01) during the swing phase. The three L5 extensor peaks after the toe-off are respectively 1.7 (*P*<0.001), 1.4 (*P*<0.001) and 1.7 (*P*<0.01) times higher for the FW group. The main results support the idea that the patterns described during steady-state gait are already observable during gait initiation. This study also provides reference data to further investigate stance limb kinetics in specific or pathologic populations during gait initiation. It will be of particular interest for elderly people, knowing that this population displays atypical trunk postures and present a high risk of falling during this forward stepping.

## Introduction

In humans, the mass of the trunk corresponds to almost half of the total body mass [1]. As a consequence, trunk position has an important impact on human motion [2], [3]. Especially, the main effects of trunk inclination on kinematics during walking are well identified, whether for able populations [4]–[6], elderly [7] or people with trunk deformities [8]. In addition, natural inclinations of the trunk with respect to the vertical, whether it be forward (FW) or backward (BW), have been highlighted during steady-state gait [2]. However, to the best of our knowledge, no study has wondered about the existence and the likely kinetic and kinematic impacts of these trunk inclinations during initiation of the gait.

Gait initiation is the process that makes it possible to place subjects in steady-state gait during the first step from an upright posture [9]. This initiation is composed of 2 successive phases: a first phase of anticipatory postural adjustments (APA) precedes and prepares the second phase of execution, with the trunk leaning forward, similar to a controlled fall [10]. Because of its imposing mass in relation to the whole body [11], controlling trunk inclination during gait initiation could appear to be challenging. This is true for able persons and mainly for specific populations, notably for elderly subjects [12] who usually display atypical trunk postures [13] and for whom the risk of falling during gait initiation is high [14]. For example, in their study, Laudani et al. [15] showed that while young and older women exhibited a forward trunk tilt during gait initiation, older women displayed specific upper body motion patterns in relation to the loss of muscle function in their lower limbs. Furthermore, Dietrich et al. [16] and Lepers & Brenière [17] reported that the subjects lean forward during the static period in order to encourage gravity's action and increase the speed of the forward-falling body while performing the first step in rapid walking. Thus, in case FW and BW leaners exist yet during gait initiation, it would be possible to hypothesize that FW leaners, predisposed to using gravity, would initiate more rapidly their gait than BW leaners.

During lateral steps, Bruyneel et al. [18] reported that the redistribution of the trunk's segmental masses observed with scoliotic adolescents [19] affected their impulse. During forward step, Azuma et al. [20] reported that the shift of the body to the stance or the swing limb, *i.e* modification of the horizontal location of the center of mass (COM), affected the duration of the gait initiation. Since, in the frontal and horizontal planes, the subject's posture influences the dynamic or kinematic characteristics of the execution of steps, it is probable that forward or backward inclination of the trunk with respect to the vertical could modify the impulse or the duration of the execution of a forward step.

During stabilized gait, different gait patterns or classes have already been highlighted [2], [21], [22], and net muscular moments are well documented for the lower limbs and the thoraco-lumbar region [2], [23], [24]. For example, Leteneur et al. [2], who described two distinct gait patterns in terms of the natural inclination of their subjects' trunks (FW or BW-leaning) during the gait cycle, also noted that net muscular moments at the hips and in the thoraco-lumbar region were higher for the BW group than the FW group at the end of the stance phase [21], [22]. From knowledge about walking, it is possible to hypothesize that, in order to initiate their gait, the FW group, predisposed to using gravity, expended a less overall muscular effort than the BW group.

Therefore, the main purposes of this study are to determine the influence of trunk's natural inclination on body kinematics and stance limb kinetics during gait initiation. More specifically, we seek to verify whether or not, during gait initiation, the trunk's natural inclination affects 1) instantaneous speed of the body's COM at the end of the first step, 2) the antero-posterior impulse of the stance limb, and 3) the stance limb's joint moments and the thoraco-lumbar moments.

## Methods

### Participants

Twenty-five healthy young males participated in this study. The average age, height, and mass were respectively 26.5±6.0 years, 1.79±0.10 m and 72.4±9.3 kg. No subjects had current back pain complaints or neurological disorders that could affect their gait initiation and trunk motion. The active range of motion was within normal limits [25] and the limb-length discrepancy, measured in the anatomical reference position between the anterior superior iliac spine and the medial malleoli, was less than 0.5 cm. The dominant lower limb, which was identified as the one the subject used to kick a ball [26], was the right limb, except for two of the subjects. Prior to the experimentation, all procedures were explained to each subject who signed a written consent. The experimental protocol was approved by the Ethics Committee of the University of Valenciennes.

### Experimental protocol

A 14-body segment model was defined by 28 spherical reflective markers, each one with a 14-mm diameter. The International Society of Biomechanics joint coordinate standards [27] were applied. For each subject, these markers were placed on the acromions, elbows, wrists and the second and fifth knuckles to define the upper limbs. The other markers were placed on the greater trochanters, mid-thigh, tibial plateaus, mid-calf, lateral malleoli, heels and the second metatarsophalangeal joints to mark the lower limbs. The head markers were located on the tragi and the glabella, and a marker was placed on the 5^th^ lumbar vertebra (L5). The neck and trunk were considered as a single segment, using shoulders and hips markers.

Before initiating gait, the subjects were standing upright, looking forward, barefoot, with the upper limbs at each side of the body. Each foot was placed on a force plate (Kistler, Switzerland). The force plates were placed side-by-side and 5 mm away from each other at the beginning of a 5 m-long walkway. Ten cameras of a Vicon 612 motion capture system were located around the force plates so as to cover a calibration volume of 2 m in length, 2 m in height, and 1.5 m in width. The sampling frequency was 120 Hz for the video and the force plates.

The subjects faced a screen placed at the end of the walkway, which served to display the visual order of gait initiation [28], appearing as a green rectangle. This visual start signal was synchronized with the motion capture system in such a way to insure an appearing event was directly detectable from the video file. The visual start signal was displayed after a 5-second period of static standing upright. The precision of this appearing event was evaluated at 1/120^th^ of a second, which was used temporally for calculating the subjects' reaction time.

Before acquiring gait initiation data, the subjects performed practice trials to familiarize themselves with the experimental conditions. They were instructed to use a natural speed and to advance their preferred lower limb as soon as they saw the visual start signal. They had to go to the end of the walkway, looking forward, with their upper limbs at the side. The subjects performed ten trials, and only the subjects who always advanced spontaneously the right lower limb were included in this study (i.e., about 80%).

Average natural trunk inclination was measured between the appearance of the visual start signal and the right heel strike, marking the end of the gait initiation [9]. Trunk inclination was defined by the angle sustained by the line connecting the midpoint between the greater trochanters and the acromion with respect to the vertical. This option has been selected for two main reasons. First, this average trunk angle was preferred to the one measured in the orthostatic position because it better reflects the actual position of the trunk and its effect on net muscular moments during gait initiation. Second, the anatomical points that are used for defining the trunk segment are determinant for its orientation in the sagittal plane. For example, Thorstensson et al. [4] have defined the trunk segment with the 7^th^ cervical vertebrae and the 3^rd^ lumbar vertebrae and reported an overall range in trunk oscillations in the sagittal plane between 1.5° and 6° during gait. With this trunk modeling, none of their 10 subjects tilted their trunk behind the vertical. In addition, Goh et al. [29], who have defined the trunk segment with the acromion processes and the anterior superior iliac spines, observed a mean backward trunk inclination of 8.4° during walking, meaning that this trunk modeling implied that no subject maintained a forward trunk tilt with respect to the vertical. It appears then that the choice of trunk modeling is very important since, depending on that, the findings could be divergent. Furthermore, Leardini et al. [30] showed that there are different patterns and ranges of motion of the trunk movements in function of its modeling. Since our study seeks to examine the trunk inclinations around the vertical, none of the two previous trunk definitions are relevant. So our trunk modeling, using the hip markers and the acromions, was mainly motivated by functional considerations.

The trunk angle was averaged for each trial and for each subject. Afterwards, the subjects were divided into 2 groups according to their average natural trunk inclinations, either backward (BW) or forward (FW), with respect to the vertical [2]. The number of subjects in each group was determined so that a statistical power of 80% was reached [31]. The average natural trunk inclination was −1.9°±1.6° for the 13 BW subjects, whereas it was 2.6°±1.6° for the 12 FW subjects. Additionally, the subjects were retested at an interval of at least three days to ensure that their natural trunk inclinations were similar.

### Data analysis

**Figure 1:**
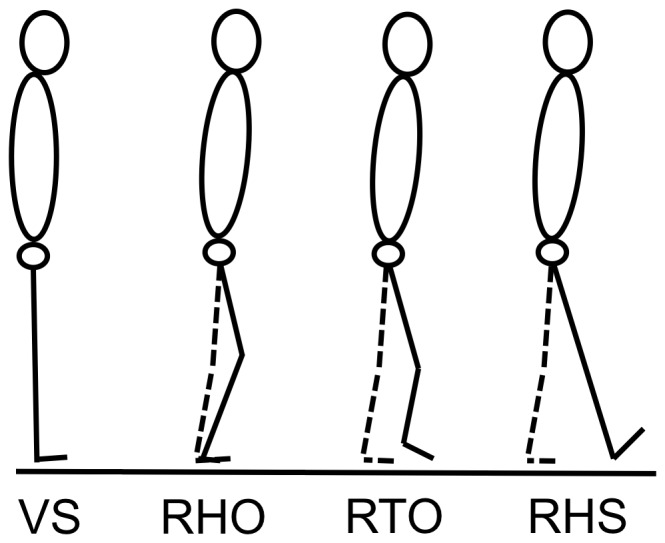
Illustration of the three kinematic events calculated from the appearance of visual signal (VS). The stance lower limb (left) is traced in dashed lines; the swing lower limb (right) is traced in solid lines. RHO: right heel off; RTO: right toe off; RHS: right heel strike.

The center of mass (COM) was calculated from the video, using the anthropometric tables of Zatsiorsky-Seluyanov modified by De Leva [1]. In addition to antero-posterior instantaneous speed of the COM at the right heel strike (RHS), four other parameters are usually used to characterize gait initiation. They were calculated from the video and force plate data. The first of these parameters is the reaction time (or the beginning of the APA) that was determined by the first variation of the vertical component of the ground reaction force [28]. The three other parameters used to characterize the global kinematics of the gait initiation of our two groups are: the instant of first knee movement (1^st^ mvt) corresponding to the lower limb segmental movement that occurred first [32], the instant of right heel off (RHO) [33]–[35] that characterizes the beginning of the execution phase and the instant of right heel strike (RHS) that characterizes the end of the swing phase (Figure 1).

For a better understanding of the net muscular moment curves, the right toe off (RTO) was also determined as a way to plot the beginning of the swing phase. In addition, the dependent spatio-temporal variables were also measured: the subjects' reaction times, calculated between the appearance of the visual start signal (VS) and the first variation of the vertical ground reaction force; the APA duration, calculated between the first variation of the vertical ground reaction force and RHO [34]; the gait initiation duration, calculated between the first variation of the vertical ground reaction force and RHS [9]; the amplitude of the backward displacement of the center of pressure (COP), calculated on the antero-posterior axis between the COP position at the first variation of the vertical ground reaction force and the maximum backward displacement of the COP.

**Figure 2:**
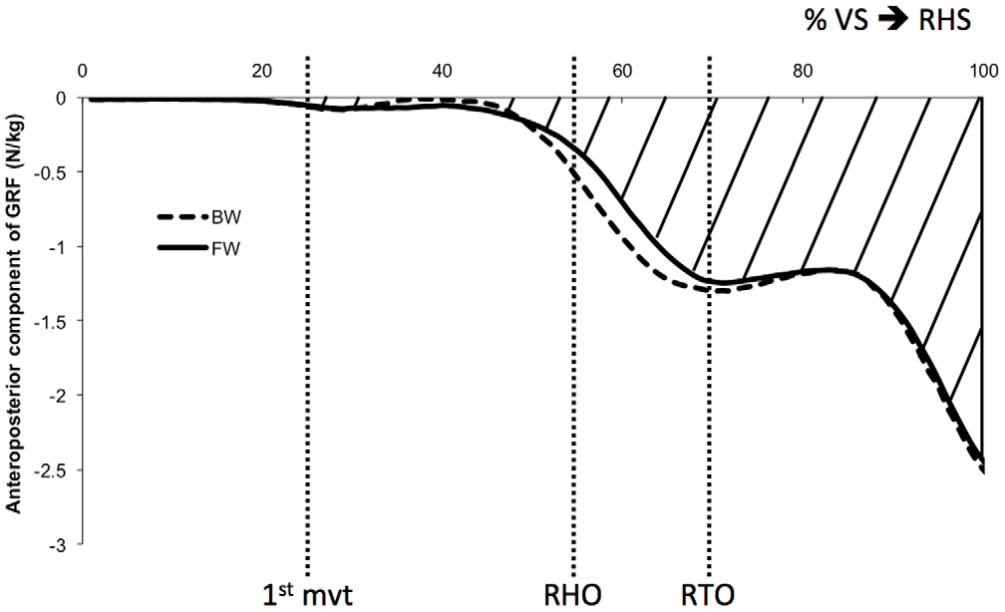
Antero-posterior component of the ground reaction force under the stance limb (left) for the BW group (dashed line) and the FW group (solid line). It was normalized temporally between the appearance of the visual signal (VS) and the right heel strike (RHS), and in amplitude in terms of the subjects' body mass. The hatched surface illustrates the antero-posterior impulse for the FW subjects, using the surface calculated under the curve between the first variation of the ground reaction force and the right heel strike. 1^st^ mvt: first knee movement (26% of the motor task); RHO: right heel off (55%); RTO: right toe off (68%). The average standard deviations for the BW and FW subjects are 0.1 N/kg. There is no significant difference between the two groups (p>0.05).

The antero-posterior impulse was calculated with the antero-posterior component of the ground reaction force under the stance lower limb (left) [36]. The forces were normalized in terms of the subjects' mass and expressed between the appearance of the visual signal (VS) and the right heel strike (RHS). However, in order to not consider the subjects' reaction times, the impulse was calculated between the first variation of the vertical ground reaction force and the RHS (Figure 2).

**Figure 3:**
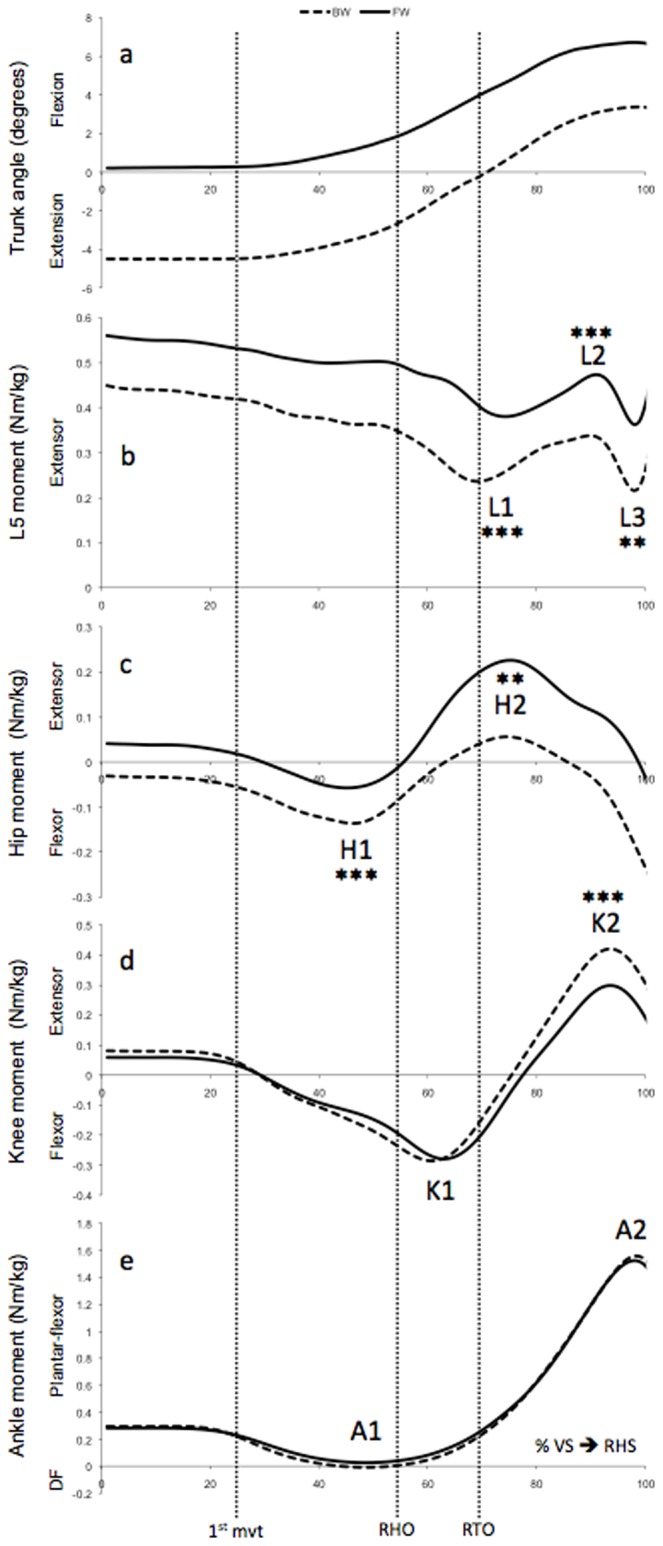
Trunk angle and lower limb joints moments for the BW group (dashed line) and the FW group (solid line). (a) Trunk angle, (b) L5 moment, (c) hip moment, (d) knee moment and (e) ankle moment, normalized in terms of the subjects' body mass, expressed in percentage of the gait initiation defined between the visual signal (VS) and the right heel strike (RHS). For the BW and FW groups, the average standard deviations are 0.1 Nm/kg for the ankle, knee, hip and L5 moments. The letters L, H, K & A respectively correspond to the L5 vertebrae, hip, knee, and ankle. 1^st^ mvt: first knee movement (26% of the motor task); RHO: right heel off (55%); RTO: right toe off (68%); DF: dorsal flexor. **: p<0.01; ***: p<0.001.

The net muscular moments for each joint of the stance lower limb and the L5 vertebrae in the sagittal plane were calculated using inverse dynamics. The inverse dynamics is based on a bottom-up method, and the angular velocity and accelerations were calculated using quaternions [37]. The amplitudes of these net muscular moments were normalized with respect to the subjects' body mass. They were also normalized temporally between the appearance of the visual signal and the first right heel strike. The curves of the ankle, knee, hip and L5 vertebrae moments were characterized by their local maximum and minimum (Figure 3). The moment peaks were labeled with an alpha-numerical code, corresponding to a letter indicating the joint and a number that increases with the duration of gait initiation. These values were used for statistical analysis.

### Statistical analysis

In order to verify the homogeneity of the subject age and the anthropometric characteristics of the two groups, a one-way ANOVA was performed on these parameters. The significance threshold was set at 5%. No significant difference was observed for these parameters between the groups (p>0.05). A one-way ANOVA was performed to determine the way that the trunk's inclination affects 1) the instantaneous speed of the body's COM at the RHS, 2) the antero-posterior impulse of the stance limb, and 3) the stance limb's joint moments and the thoraco-lumbar moments during gait initiation. The ANOVAs were followed by Bonferroni post-hoc tests for p<0.05. Finally, Cohen's d coefficient was calculated for statistically significant parameters to estimate size effect. The threshold was set at 0.8 [31], [38].

## Results

### Kinematic and spatio-temporal parameters

In general, as indicated by the patterns of the curves in Figure 3A, the natural inclination of the trunk in the sagittal plane varies similarly for both groups during gait initiation, but with an off-set of nearly 4°. In the period that goes from the 1^st^ knee movement to the right heel strike (i.e., from around 26% to 100% of gait initiation), all subjects lean forward, around 7° on average.

First, the 1^st^ knee movement (1^st^ mvt) occurs, at an average of 26%±3% of the motor task (i.e., 332 ms±73 ms after the appearance of the visual signal (VS)). In 90% of cases, it is the left knee that moves first. The interval between the 1^st^ movements of the left and right knee is about 17 ms. The right heel off (RHO) occurs at 55%±4% of the gait initiation, (i.e., 692 ms±120 ms after VS). Finally, the right heel strike (RHS), which characterizes the end of gait initiation, occurs at an average of 1290 ms±110 ms after VS. Considering the values expressed in percents of the motor task duration, the difference in the mean values of both BW and FW groups do not exceed 1 point. In terms of the instantaneous speed of the body COM according to the antero-posterior axis at the RHS, there is no significant difference between the BW group and the FW group (F(1.23) = 0.57; *P*>0.05) (Table 1). Table 1 provides the four other parameters (1^st^ mvt, RHO, RHS, duration of APA, reaction time), which were calculated to characterize gait initiation of the BW and FW groups. Both groups do not have significant differences for these parameters (F(4.20) = 1.15; *P*>0.05).

**Table 1:** Age, height, mass, kinematic and spatio-temporal parameters for the subjects in the Backward (BW) and Forward (FW) groups (mean±standard deviation).

	BW (13 subjects)	FW (12 subjects)
Age (years)	26.2±7.1	26.9±4.8
Height (m)	1.79±0.10	1.79±0.10
Mass (kg)	69.0±5.5	76.0±11.3
1^st^ knee movement (ms)	318±81	355±65
Right Heel Off (RHO) (ms)	662±117	745±93.3
Right Heel Strike (RHS) (ms)	1250±125	1333±100
Reaction Time (ms)	287±80	316±79
APA duration (ms)	375±82	429±66
Gait initiation duration (ms)	961±90	1017±68
Backward COP shift (cm)	5.3±0.8	4.9±1.1
COM speed at the RHS (m/s)	1.00±0.08	0.98±0.05

The spatio-temporal parameters were calculated from the appearance of the visual start signal. There is no significant difference between the two groups (p>0.05). APA: anticipatory postural adjustments. COP: center of pressure COM: center of mass.

### Impulse

Figure 2 shows the antero-posterior component of the ground reaction force for the stance limb (left). This component was used to calculate the antero-posterior impulse. There is no significant difference between the BW subjects (−0.83 N.s/kg±0.06 N.s/kg) and the FW subjects (−0.82 N.s/kg±0.06 N.s/kg) (F(1.23) = 0.03; *P*>0.05). These values are lower than the antero-posterior final COM velocity (Table 1) because only the antero-posterior component of ground reaction force of the stance lower-limb (left) was used to calculate the impulse. Mechanically, the value of the impulse per unit of mass should be the same as the one of the final COM velocity. When using the antero-posterior component of ground reaction force of the two lower limbs, it appears that the antero-posterior impulse (1.00 N.s/kg±0.08 N.s/kg for the whole population) is indeed similar (p = 0.86, df = 48) to the antero-posterior final COM velocity (0.99 m/s±0.07 m/s for the whole population), as expected from the classical mechanics.

### Muscular moments

In general, the net muscular moments of the FW group show a pattern similar to the BW group, as shown in Figure 3. There is no significant difference for the two groups in terms of the ankle moments calculated at peaks A1 (*P*>0.05; df = 23) and A2 (*P*>0.05; df = 23) (Figure 3E). For the knee, there is also no significant difference between the BW and FW subjects in terms of flexor peak K1 (*P*>0.05; df = 23) (Figure 3D). However, just before the RHS, the knee's extensor moment K2 (Figure 3D) for the BW group (0.47 Nm/kg±0.06 Nm/kg) is 1.4 times higher (*P*<0.001; df = 23) than for the FW group (0.34 Nm/kg±0.10 Nm/kg). At the hip, before the RHO, the flexor moment H1 of the BW group (−0.16 Nm/kg±0.04 Nm/kg) is around 2.4 times higher (*P*<0.001; df = 23) than the one for the FW group (−0.07 Nm/kg±0.07 Nm/kg) (Figure 3C). The extensor moment H2, which occurs a bit after the right toe off, is around 3.1 times higher (*P*<0.01; df = 23) for the FW group (0.26 Nm/kg±0.16 Nm/kg) than for the BW group (0.08 Nm/kg±0.15 Nm/kg).

For the 5^th^ lumbar vertebrae, at the peak L1, the extensor moment for the FW group (0.36 Nm/kg±0.07 Nm/kg) is around 1.7 times higher (*P*<0.001; df = 23) than the one for the BW group (0.21 Nm/kg±0.09 Nm/kg) (Figure 3B). At the peak L2, the extensor moment for the FW group (0.49 Nm/kg±0.08 Nm/kg) is around 1.4 times higher (*P*<0.001, df = 23) than the one for the BW group (0.36 Nm/kg±0.04 Nm/kg). At the peak L3, the extensor moment for the FW group (0.34 Nm/kg±0.09 Nm/kg) is around 1.7 times higher (*P*<0.01; df = 23) than the one for the BW group (0.20 Nm/kg±0.11 Nm/kg). The average Cohen's d coefficient was 1.54 for all statistically significant lower limb joint moments.

## Discussion

This study sought to determine whether or not the FW or BW natural inclinations of the trunk influence body kinematics and stance limb kinetics during gait initiation. The primary result is that only the net muscular moments are affected by the trunk's natural inclination during gait initiation, for similar temporal characteristics. To the best of our knowledge, this study is the first to have investigated the muscular moments of the stance limb during gait initiation.

The backward shift of the COP and the speed of the COM calculated in this study during gait initiation were similar to those described by Brenière et al. [35]. The trajectory of the COP for the FW subjects is not statistically different from the COP trajectory for the BW subjects. In addition, Lepers & Brenière [17] highlighted the relationship between trunk inclination and gait initiation speed: high speeds were characterized by a more important degree of forward flexion of the trunk. However, in this study, since the COM speed at right heel strike (i.e., at the end of gait initiation) was not statistically different between the BW and FW groups, the postural differences of the trunk during gait initiation could not be attributed to different walking speeds between the two groups. Furthermore, the forward flexion of the trunk during gait initiation (Figure 3A) for the BW and FW groups evolves similarly to the results reported by Laudani et al. [15] for comparable-aged subjects. Moreover, the natural inclination of the trunk, forward or backward, does not affect the spatio-temporal characteristics or the antero-posterior impulse of the stance limb. Thus, both the BW and FW seem to initiate their gait identically. This result has to be put in relationship with the stereotypical and robust nature of the process [16], [34], [35], [39]–[42]. In our study, the lack of significant differences for the spatio-temporal parameters and the antero-posterior impulse, despite the different trunk postures during gait initiation, led us to wonder whether the net muscular moments could eventually be impacted by the natural trunk inclination.

Depending on the trunk's natural inclination, forward or backward, the net muscular moments developed in the lower limbs were actually significantly different during gait initiation. It has been already established that the natural inclination of the trunk affects the net muscular moments of the stance limb and of the L5 vertebrae during steady-state gait [2]. In their study, Leteneur et al. [2] showed that the BW group propelled themselves with a strong hip flexor moment, while the FW group used their hip extensors throughout stance.

In the present study, before the heel off (HO) of the swing limb, both groups display a hip flexor peak in the stance limb (Figure 3C), which could explain the trunk's forward tilt [17]. The difference observed between the FW subjects and the BW subjects at peak H1 could be linked to the fact the FW subjects profit from the gravity action to propel themselves [17]. Thus, they display less muscular actions at the hip than the BW subjects. However, during the swing phase, the difference observed at peak H2 revealed that the FW subjects need to display a hip extensor moment to control their trunk's forward inclination [2], [23].

In this study, all of the subjects tilted the trunk forward during gait initiation until the toe off (TO), which is related to a gradual reduction of the lumbar (L5) extensor moments (Figure 3A and 3B). These lumbar extensor moments could respond to the need to control the trunk's forward tilt. However, when the subjects toe off with the swing limb, they continue to tilt the trunk forward; these lumbar extensor moments increase and can reach the peak L2. The increase of these moments may be related to the appearance of a hip extensor peak during this swing phase (Figure 3C). This observation suggests the need of controlling both the trunk's inclination and the forward swing of the lower limb. Furthermore, by highlighting the relative contribution of each body segment at the genesis of the propulsive forces during steady-state gait, Gillet et al. [3] showed that, because of their higher mass, the movements of the trunk and the swing limb thigh contribute the most to the forward propulsion. Moreover, the FW group, who leant the trunk more forward, displayed higher L5 and stance hip moments. These results are comparable to those found during steady-state gait for the FW and BW trunk inclinations [2]. Thus, the trunk's natural inclination, forward or backward, led to different gait initiation strategies.

During the swing phase (i.e., from TO to RHS), both groups display variations in the knee and ankle moments for the stance limb (Figure 3D and 3E). For both groups, the knee extensor peak K2 can be compared to the one observed during steady-state gait in order to control the collapse of the stance limb at the heel strike of the swing limb [24]. The trunk's forward inclination in front of the vertical could perhaps explain that the FW subjects use a knee extensor moment K2 significantly smaller than the BW subjects. In this study, the variations of plantar-flexor moment developed by the stance ankle (Figure 3E) reflect the motor activity traditionally described for the ankle during gait initiation. Until the heel off (HO), these moment's variations are related to the anticipatory postural adjustments (APA) characterized, from a motor perspective, by an inactivation of the plantar-flexor muscles and an activation of the dorsal-flexor muscles of the ankle [33], [35], [39]. From the HO to the RHS (i.e., during the execution phase [34]), the increase in the ankle's plantar-flexor moment is essentially a moment that controls the dynamic postural balance by limiting the body's forward fall [43], [44]. While the trunk's natural inclinations affect the lumbar (L5) moments and the hip and knee moments of the stance limb, they do not affect the ankle moments.

## Conclusions

The natural trunk inclinations, forwards or backwards, affect the joint moments of the stance limb at the hip and knee during gait initiation. However, it does not affect the COM speed at the first heel strike or the antero-posterior impulse of the stance limb. It appears that these differences could be connected to the different gait initiation patterns, in which the FW subjects profit from gravity to propel themselves, while the BW subjects lean forward using their hip flexor muscles more. These differences do not hide the need of all the subjects to use control moments at the level of the L5 vertebrae and the knee during the swing phase of the lower contralateral limb. The value of the control moments depends on the trunk's natural inclination during gait initiation. These results support the idea that the characteristics of the steady-state gait kinetics are already observable during gait initiation.

The methodology used in the present study may be reinvested in further studies about gait initiation in specific or pathologic populations, such as in people who are at risk of falling or in chronic low back pain sufferers. Moreover, the present data may be used as reference in these future studies.
